# Protein overabundance is driven by growth robustness

**Published:** 2024-08-21

**Authors:** H. James Choi, Teresa W. Lo, Kevin J. Cutler, Dean Huang, W. Ryan Will, Paul A. Wiggins

**Affiliations:** 1 Department of Physics, University of Washington, Seattle, Washington 98195, USA; 2 Department of Laboratory Medicine and Pathology, University of Washington, Seattle, Washington 98195, USA; 3 Department of Microbiology, University of Washington, Seattle, Washington 98195, USA; 4 Department of Bioengineering, University of Washington, Seattle, Washington 98195, USA

## Abstract

Protein expression levels optimize cell fitness: Too low an expression level of essential proteins will slow growth by compromising essential processes; whereas overexpression slows growth by increasing the metabolic load. This trade-off naïvely predicts that cells maximize their fitness by sufficiency, expressing just enough of each essential protein for function. We test this prediction in the naturally-competent bacterium *Acinetobacter baylyi* by characterizing the proliferation dynamics of essential-gene knockouts at a single-cell scale (by imaging) as well as at a genome-wide scale (by TFNseq). In these experiments, cells proliferate for multiple generations as target protein levels are diluted from their endogenous levels. This approach facilitates a proteome-scale analysis of protein overabundance. As predicted by the Robustness-Load Trade-Off (RLTO) model, we find that roughly 70% of essential proteins are overabundant and that overabundance increases as the expression level decreases, the signature prediction of the model. These results reveal that robustness plays a fundamental role in determining the expression levels of essential genes and that overabundance is a key mechanism for ensuring robust growth.

Understanding the rationale for protein expression levels is a fundamental question in biology with broad implications for understanding cellular function [1]. Measured expression levels appear to be paradoxically both *optimal* and *overabundant*. For instance, repeated investigations support the idea that gene expression levels optimize cell fitness [2, 3]. Since the overall metabolic cost of protein expression is large [4, 5], fitness optimization would seem to imply that protein levels should satisfy a *Goldilocks condition*: Expression levels should be *just high enough* to achieve the required protein activity [6, 7]. However, a range of approaches suggest that many essential genes are expressed in vast excess of the levels required for function [7–9]. How can expression levels be at once optimal with respect to fitness as well as in excess of what is required for function?

The cell faces a complex regulatory challenge: Even in a bacterium, there are between five and six hundred essential proteins, each of which is required for growth [10]. How does the cell ensure the robust expression of each essential factor? We recently argued that the stochasticity of gene expression processes fundamentally shape the principles of central dogma regulation, including the optimality of protein overabundance [11]. Specifically, we proposed a quantitative model, the Robustness-Load Trade-Off (RLTO) model, which makes a parameter-free prediction of protein overabundance as a function of gene transcription level [11]. The optimality of overabundance can be understood as the result of a highly-asymmetric fitness landscape: the fitness cost of essential protein underabundance, which causes the arrest of essential processes, is far greater than the fitness cost of essential protein overabundance, which leads to slow growth by increasing the metabolic load. However, critical model assumptions and predictions remain untested which is the motivation for the current study. Here, we will quantitatively measure the fitness landscape with repect to protein abundance and determine the level of overabundance for all essential proteins in the bacterium *Acinetobacter baylyi*.

## RESULTS

### Natural competence facilitates knockout-depletion.

**FIG. 1.**
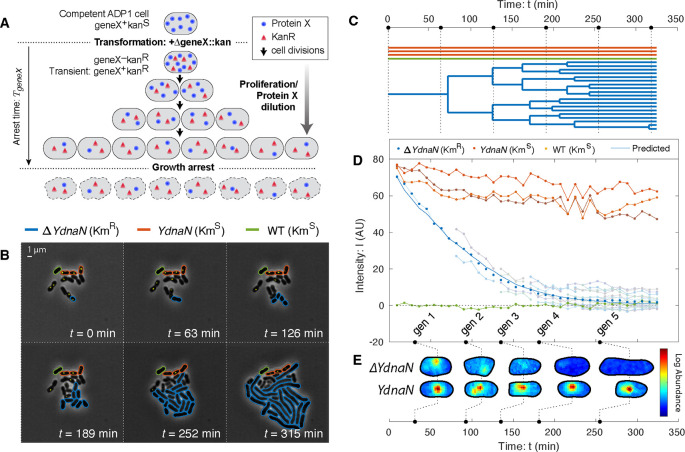
Knockout-depletion experiments. **Panel A: Experimental schematic.** Competent ADP1 cells are transformed with Δ*geneX::kan*. Untransformed cells arrest immediately on selective media. Transformed cells proliferate, but cease protein X expression (blue circles) while expressing Kan (red triangles). Existing protein X abundance is diluted as cells proliferate. For essential genes, cell growth continues until protein levels are diluted to the threshold level required for growth, after which growth arrests. **Panel B & C: Visualization of knockout depletion.** The fluorescent fusion *YPet-dnaN* to essential gene *dnaN* is knocked outed at *t* = 0. Cell proliferation is visualized using phase-contrast microscopy while protein abundance is measured by fluorescence microscopy (yellow). Transformed cells (Δ*YdnaN*, blue) have a Km^*R*^ allele and can proliferate over several generations before arrest; however, untransformed cells (*YdnaN*, orange) and wild-type cells (WT, green) were both kanamycin sensitive and therefore arrested immediately. **Panel C: Lineage tree.** Black dotted lines represent time points shown in Panel B. **Panel D: Target protein is diluted by proliferation.** Protein concentration is measured by integrated fluorescence. Arrested *YdnaN* cells maintain protein abundance, whereas proliferating transformed cells (Δ*YdnaN*, blue) show growth-induced protein depletion. The protein concentration over all transformed progeny (blue points) are consistent with the dilution-model prediction (solid blue). **Panel E: Protein function is robust to dilution.** Representative single-cell images of transformed (Δ*YdnaN*) and untransformed (*YdnaN*) cells are shown for successive time points. The YPet-DnaN fusion shows punctate localization, consistent with function, even as protein abundance is depleted. No puncta are observed in the last generation and the cells form filaments, consistent with replication arrest.

To characterize the fitness landscape for essential gene expression, we must deplete the levels of essential proteins. Both degron- and CRISPRi-based approaches have been applied; however, these approaches require careful characterization of protein levels [8, 12–15] and introduce significant cell-to-cell variation on top of the endogenous noise which further obscures the underlying fitness landscape [16]. To circumvent these difficulties, we will use an alternative approach: *knockout-depletion* in the naturally competent bacterium *A. baylyi* ADP1 [17, 18]. In this approach, cells are transformed with a *geneX::kan* knockout cassettes targeting essential gene X, carrying a kanamycin resistance allele Km^*R*^. (See Fig. 1A.) Cells that are not transformed arrest immediately on selective media. The crux of the approach is that transformants remain transiently *geneX*^+^, due to the presence of already synthesized target protein X, even after the transcription of the target *geneX* stops. Growth can continue, diluting protein X abundance, as long as this residual abundance remain sufficient for function. The success of the knockout-depletion approach is dependent on the extremely high transformation efficiency of *A. baylyi*.

### Target proteins are depleted by dilution.

A key untested assumption in the experimental design of the knockout-depletion approach is that target protein translation stops after transformation, and that the protein abundance is depleted by dilution. The model predicts that the protein concentration is:

(1) $$C(t)=C_{0}\cdot V_{0}/V(t),$$

where $C_{0}$ and $V_{0}$ are the concentration and volume of the progenitor cell at deletion and V(t) is the total volume of the progeny. To test the predicted protein depletion hypothesis, we designed a knockout-depletion experiment to target a protein we had previously studied that can be visualized using a fluorescent fusion and whose localization is activity dependent: the essential replication gen*e dnaN, whose gene product is* the β sliding clamp [19–21]. We constructed a N-terminal fluorescent fusion to *dnaN* using YPet in *A. baylyi* at the endogenous locus. The resulting mutant (YdnaN) had no measurable growth defect under our experimental conditions. We then knocked out the *YPet-dnaN* fusion, yielding Δ*dnaN*, and characterized the protein levels by quantifying YPet-DnaN abundance by fluorescence. The experimentally measured fluorescence intensity is consistent with the dilution model (Eq. 1), as expected. (See Fig. 1D.) We therefore conclude that knockout-depletion experiments are consistent with the experimental design shown schematically in Fig. 1A.

### Replication persists during DnaN depletion.

A key subhypothesis of the overabundance model for transient growth is that target protein function continues as the target protein abundance is depleted. An alternative hypothesis for transient growth of the Δ*dnaN* strain is a high initial chromosomal copy-number that is partitioned between daughter cells, even after the replication process itself arrests due to target protein depletion [4, 22]. The imaging-based knockout-depletion experiment tests this hypothesis as well. The localization of DnaN is dependent on activity: During ongoing replication, DnaN is localized in puncta corresponding to replisomes, whereas in the absence of active replication, DnaN has diffuse localization [19–21, 23, 24]. During the knockout-depletion experiment, we observed YPet-DnaN puncta persist as the targeted fusion was depleted (Fig. 1DE), consistent with replication activity after dilution. Only after the YPet-DnaN puncta disappear do the cells begin to adopt the Δ*dnaN* phenotype: cell filamentation (Fig. 1BE). We therefore conclude that function (replication) is robust to significant target protein (DnaN) dilution.

### Many essential knockouts undergo transient growth.

**FIG. 2.**
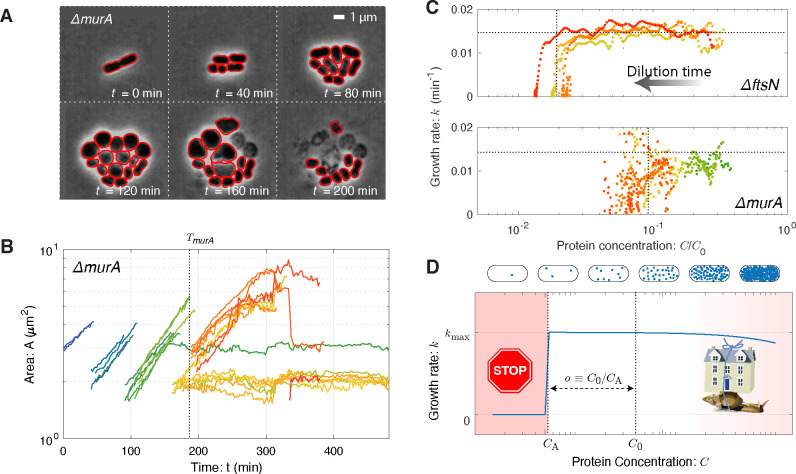
The fitness landscape. **Panel A: Visualization of growth in a *murA* knockout.** Essential gene *murA* is knocked out at t=0 and cell proliferation is visualized by phase-contrast microscopy. Red outlines represent the Omnipose cell segmentation. Cell proliferation continues for multiple generations after deletion. **Panel B: Quantitative analysis of cell proliferation with single-cell resolution.** Cell area (log scale) as a function of time for the *murA* deletion. The log-slope represents the single-cell growth rate. The vertical dotted line represents the arrest time at which cell growth slows to cell arrest. **Panel C: Growth rate as a function of protein depletion for** Δ***ftsN* and** Δ***murA*.** In both essential gene deletions, the growth rate is observed to obey the step-like-dependence, transitioning between wild-type growth to arrest at the vertical dotted lines. We define the critical dilution as $o\equiv C_{0}/C_{A}$ where $C_{A}$ is the protein concentration at arrest. **Panel D: The fitness landscape is threshold-like.** Motivated by single-cell growth data, cell fitness is modeled using the Robustness-Load Trade-Off model (RLTO). In the model, there is a metabolic cost of protein expression which favors low expression; however, growth arrests for protein concentration C smaller than the threshold level $C_{A}$ (red). The relative metabolic cost of overabundance is small relative to the cost of growth arrest due to the large number of proteins synthesized, resulting in a highly asymmetric fitness landscape [11].

To understand the generic consequences of essential protein depletion, we used the imaging-based knockout-depletion experiments to explore essential genes with a range of functions. We initially targeted four essential genes: the replication initiation regulator gene *dnaA* (movie), the beta-clamp gene *dnaN* (movie), the cell-wall-synthesis gene *murA* (movie), and septation-related gene *ftsN* (movie), as well as a non-essential IS element with no phenotype as a negative control (movie). (Representative frame mosaic images and cytometry appear in Supplementary Material Sec. 4 D.) In each case, transformants continued to proliferate through multiple cell-cycle durations [17] and are therefore consistent with the essential protein overabundance hypothesis. However, in Ref. [17], we were unable to perform a quantitative single-cell analysis of these time-lapse experiments since existing segmentation packages failed to segment the observed morphologies [25]. We therefore developed the *Omnipose* package, which facilitated quantitative analysis of the growth dynamics with single-cell resolution [25]. (See Fig. 2A.)

### The fitness landscape is threshold-like.

A key input to the RLTO model is the fitness landscape (growth rate) as a function of protein abundance. Omnipose segmentation facilitates the measurement of single-cell growth rates from the time-lapse imaging experiments. We focus first on the single-cell areal growth rate:

(2) $$k\left(t\right)=\frac{d}{dt}\ln A\left(t\right),$$

where A(t) is the area of the cell at time t. This areal growth rate is more convenient than a cell-length based rate since we avoid the necessity of defining cell length for unusual cell morphologies like those observed in the Δ*murA* mutant. Fig. 2B shows representative knockout-depletion dynamics of cell area for the essential-gene target *murA*. The log slope remains constant for multiple generations, consistent with a constant growth rate, even as the gene targeted is depleted over multiple cell cycles. By combining the dilution model (Eq. 1) and the growth rate (Eq. 2), a single knockout-depletion measurement determines the growth rate for a range of protein abundances between wild-type abundance and those realized at growth arrest. This fitness landscape is shown for the MurA and FtsN proteins in Fig. 2C. For all four mutants, the areal growth rate is roughly constant for multiple generations before undergoing a rapid transition to growth arrest.

### Protein overabundance.

We will define the overabundance as the ratio of protein concentration in wild-type cells $\left(C_{0}\right)$ to the concentration at cell arrest $\left(C_{A}\right)$:

(3) $$o\equiv C_{0}/C_{A},$$

as shown in Fig. 2D. (Supplementary Material Sec. 4 gives a detailed description of the inferred overabundance from single-cell data.) The measured overabundance for the four mutants imaged by microscopy is summarized in Tab. I, using three distinct metrics for growth. We conclude that for each gene, with the exception of *dnaA*, rapid growth continues after the knockout due to the vast overabundance of the target protein.

### The RLTO model predicts protein overabundance.

The RLTO model explicitly analyzes the trade-off between growth robustness to noise and metabolic load and predicts the optimal central-dogma regulatory principles [11]. Critically, the model incorporates the observed threshold-like dependence of growth rate on protein abundance (Fig. 2CD). The model quantitatively predicts protein overabundance with a signature feature: high-expression genes have low protein overabundance (o≈1) due to the high metabolic cost of increasing expression and low inherent noise of high expression genes; however, low-expression genes have high overabundance (o≫1) due to the low metabolic cost of increasing expression and the high inherent noise of low expression genes. (See Supplemental Material Sec. 7 for a more detailed description of the model.)

### TFNseq determines overabundances genome-wide.

**FIG. 3.**
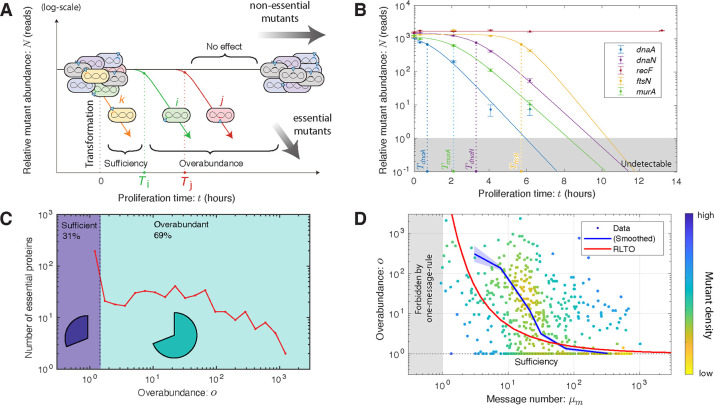
A proteome-wide analysis of protein overabundance. **Panel A: TFNseq schematic.** A poly-clonal library of knockout mutants is generated by the transformation of ADP1 with DNA mutagenized by transposon insertions. The library is proliferated on selective media and sequential fractions are collected. The relative-abundance trajectories of mutants are determined by mapping transposon insertion sites by sequencing. **Panel B: TFNseq-trajectory analyses for five mutant strains.** Each mutant trajectory is well fit by one of the three trajectory models. As expected, the no-effect model is selected for the non-essential gene *recF*. For the other four essential genes, the overabundance model is selected. The dotted line represents the arrest time for each mutant. **Panel C: Overabundance varies by orders of magnitude between essential proteins.** The protein overabundance is inferred from the arrest time using Eq. 4. Sufficient expression genes have overabundance o=1, while overabundant genes vary from o>1 to very large overabundance (o>100). **Panel D: Overabundance is large for low-expression essential proteins.** The measured message-number-overabundance pairs are shown for essential genes (including estimated gene density.) The smoothed experimental data is shown in blue (with experimental uncertainty.) The RLTO model (red) predicts that overabundance grows rapidly as the transcription level is reduced. The RLTO model qualitatively captures the trend of the data (blue); however, it appears to underestimate the measured overabundance for intermediate expression genes.

To test the signature expression-dependent overabudance prediction of the RLTO model, we now transition to a genomic-scale analysis. The Manoil lab developed a TFNseq-approach to knockout-depletion experiments for targeting all genes simultaneously in *A. baylyi* [18]. In short: A genomic library was prepared and mutagenized using a transposon carrying the Km^R^ allele. The resulting DNA was then transformed into *A. baylyi*. The transformants were propagated on selective liquid media and fractions collected every two hours from which genomic DNA was extracted. The transposons were then mapped using Tn-seq to generate the relative abundance trajectory for each mutant [18]. (See Fig. 3AB.) We then analyzed each mutant trajectory statistically using three competing growth models: no-effect, sufficiency, and overabundance, using two successive null-hypothesis tests. (See Supplementary Material Sec. 5.) For each mutant i described by the overabundance model, the TFNseq experiment measures a growth arrest time $T_{i}$ and the corresponding target protein overabundance:

(4) $$o_{i}=\exp\left(k_{0}T_{i}\right),$$

where $k_{0}$ is the wild-type growth rate. (See Supplementary Material Sec. 5.)

To test the consistency of this TFNseq approach with imaging-based knockout-depletion measurements, we focused first on the analysis of the mutants *dnaA*, *dnaN*, *ftsN*, and *murA*. As shown in Fig. 3B, the trajectories for *dnaA*, *murA*, *ftsN*, and *dnaN* show an unambiguous steplike change in growth dynamics: The no-effect trajectory model (null hypothesis) are rejected with p-values that are below machine precision, and the sufficiency trajectory model is also rejected with *p* < 10^−4^ for all genes. In Tab. I, we compare protein overabundances determined by imaging- and sequencing-based approaches. These numbers are qualitatively consistent. For instance, the single-cell analysis of *dnaA* mutant shows a nearly immediate phenotype by imaging (*i.e.* cell filamentation). (See Supplementary Sec. 4 D 3.) Likewise, the TFNseq-approach finds an overabundance of 1.0, meaning that protein expression is sufficiency. On the other hand, all three of the other mutants (*murA*, *ftsN*, and *dnaN*) are found to have very large overabundances, and are roughly comparable. Finally, a representative non-essential gene (*e.g. recF*) shows no effect. These results support the use of the TFNseq approach to analyze protein overabundance genome wide.

### Many essential proteins have vast overabundance.

To determine the protein overabundance genome-wide, we analyzed the knockout-depletion trajectories for all genes in *A. baylyi*. (See Fig. 3BCD.) Our analysis showed that the vast majority (90%) of genes annotated as non-essential were classified as having *no effect* and 10% of non-essential genes had measurable growth defects. (See Supplementary Material Fig. S10.) The most severe growth defect in non-essential annotated genes were observed for the genes *gshA* and *rplI*. For essential genes, all mutants were observed to have growth defects, as anticipated; however, only 31% of essential proteins were classified as *sufficient*, corresponding to an immediate change in growth rate. Notable genes in this category include ribosomal proteins RpsQ and RpsE, ribonucleotide reductase subunits NrdA and NrdB, and ATP synthase subunits AtpA and AtpD. However, as predicted by the RLTO model, the majority of essential proteins (69%), were classified as *overabundant*, meaning that they required significant dilution before a growth rate change was detected. Fig. 3D shows a histogram of essential gene overabundances.

### Low-expression genes are highly overabundant.

To understand the overall significance of overabundance in a typical biological process, we determined the median essential protein overabundance: 7-fold. To understand the significance of overabundance from the perspective of the metabolic load, we also determine the mean protein overabundance, weighted by the expression level: 1.6-fold. These two superficially-conflicting statistics emphasize a key predicted regulatory principle: overabundance is high for low-abundance proteins; however, it is close to unity for the high-abundance proteins, which constitute the dominant contribution to the metabolic load.

To explicitly test the predicted relation between protein expression and overabundance, we measured the relative abundance of mRNA messages by RNA-Seq for exponentially growing *A. baylyi* cells. (See Supplementary Material Sec. 6 C.) We computed the message number (transcripts per gene per cell cycle) for each essential gene. (See Supplementary Material Sec. 6 B.) Fig. S9 compares measured message numbers and overabundances for all essential genes with the prediction of the RLTO model.

As predicted, the data shows a clear trend of decreasing overabundance with increasing message number (Fig. 3D). To quantitatively capture this trend, we computed the mean log overabundance over windows of message number (blue curves) to compare the data cloud to the RLTO model predictions. With very few exceptions, high expression genes have extremely low overabundance. At the other extreme, low expression genes typically have large to very large overabundance as shown by the sharp up-turn of the blue curve as the message number approaches the one-message-rule threshold, a lower threshold on transcription that we recently proposed [11].

## DISCUSSION

### The shape of the fitness landscape.

Despite some large-scale measurements [8, 9, 26, 27], fundamental questions remain about the structure of the fitness landscape and its rationale [7]. Our measurements reveal that most (69%) essential proteins show a step-like transition between wild-type and arrested growth below a critical threshold protein abundance. Although asymmetric landscapes have been observed previously (*e.g.* [3, 26]), the knockout depletion approach is expected to yield more quantitative results. For instance, the use of either CRISPRi (*e.g.* [8]) or inducible promoters (*e.g.* [3]) significantly increases the cell-to-cell variation in protein abundance [16, 28], obscuring the features of the fitness landscape. The sharpness of the protein-abundance threshold is manifest in the single-cell analysis where the progeny begin from a common pool of protein in a single progenitor cell and are therefore not subject to noise (*e.g.* Fig. 2C).

### The rationale for a threshold abundance.

**FIG. 4.**
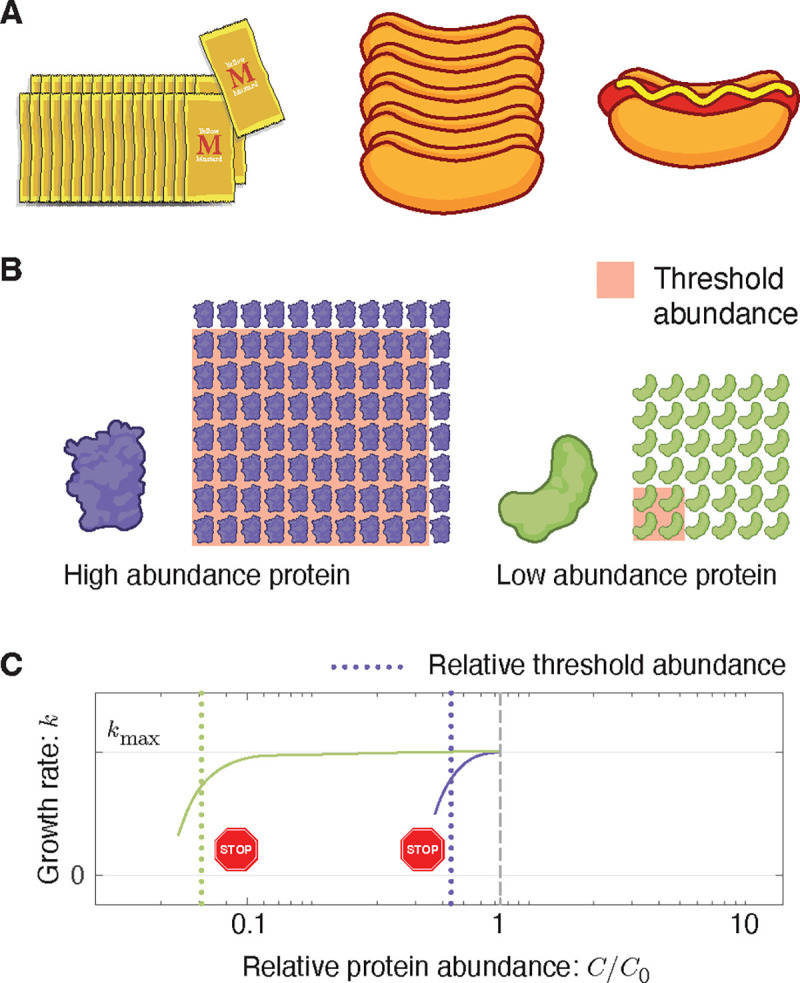
How rate-limited kinetics shapes the fitness landscape. **Panel A: An analogy for rate-limited kinetics.** The number of sausage sandwiches assembled from the pictured ingredients is limited by a single ingredient, the sausages. A depletion of either bun or mustard abundance does not immediately affect the sandwich number. **Panel B: Protein abundance and threshold.** Two essential protein species with different abundances are pictured schematically. The threshold abundance at which each protein becomes limiting is represented by the pink square and the total cellular abundance is represented by the protein array. **Panel C: Emergent fitness landscape.** A schematic model of the growth rate versus relative protein abundance is shown for the two protein species. The RLTO model predicts that low-abundance proteins (green) have high overabundance, which leads to significant insensitivity to protein depletion. High-abundance protein (purple) are predicted to have small overabundance leads to high sensitivity to protein dilution. The growth rate rapidly decreases with concentration once a species becomes limiting.

The observed threshold-like dependence can be rationalized in terms of chemical kinetics: If the protein target is not a rate-limiting reactant in an essential cellular process, then its depletion has no effect on the rate [11, 29]. See Fig. 4. We explicitly demonstrate protein function (*i.e.* replication) is robust to an order-of-magnitude depletion of replisome protein DnaN; however, for most proteins, we must infer this picture from the growth rate.

### The rationale for overabundance.

Rate-limiting kinetics does not in itself predict vast protein overabundance. The RLTO model predicts that this feature of the fitness landscape is a consequence of a balance between (i) the metabolic cost of protein expression, which favors minimizing protein abundance, and (ii) robustness to the noise in gene expression [30, 31]. The model predicts expression-dependent protein overabundance: large overabundance for low-abundance proteins and small overabundance for high-abundance proteins [11]. We show that this signature prediction is observed (Fig. 3D). In spite of predicting the genomic-scale trend, there are some significant outliers. We discuss their significance as well as evidence for the conservation of overabundance in Supplementary Material Sec. 1

### Biological implications.

Many important proposals have been made about the biological implications of noise [32]. Our work reveals that noise acts to inflate the optimal expression levels of low-expression proteins and, as a result, significantly increases the metabolic budget for protein, which constitutes 50–60% of the dry mass of the cell [4]. We believe this increased protein budget has cellular-scale implications. For instance, in stress response and stationary phase, the presence of a significant reservoir of overabundant protein provides critical resources, via protein catabolism, to facilitate the adaptation to changing conditions [33, 34]. Protein overabundance may have important implications for individual biological processes as well, including determining which proteins and cellular processes make attractive targets for small molecule inhibitors (*e.g.* antibiotics) [27]. Since overabundance defines the fold-depletion in protein activity required to achieve growth arrest, high-overabundance proteins are predicted to be extremely difficult targets for inhibition.

### Conclusion.

By combining imaging-, genomic-, and modeling-based approaches, we provide a both a quantitative measurement of the fitness landscape for all essential proteins as well as a clear qualitative and conceptual understanding of the rationale for the observed fitness landscape. The RLTO model fundamentally reshapes our understanding of the rationale for protein abundance. The model predicts, and experiments confirm, that low-abundance proteins are expressed in vast excess of what is required for growth. Despite the limitations of the experiments, the predicted trend is clearly resolved both at a genomic-scale, using sequencing-based approaches, as well as at the single-cell scale, as observed by microscopy. The rationale for the overabundance strategy is intuitive: Growth requires the robust expression of between five to six hundred distinct proteins. The cell contends with this extraordinary complex regulatory challenge by keeping all but the highest-abundance proteins in vast excess.

## Supplementary Material

Supplement 1

## Figures and Tables

**Table 1:** Measured overabundance for sequencing- versus imaging-based approaches. The overabundance was determined by both sequencing- and imaging-based approaches. For the imaging-based approach, we show two measurements based on different metrics for arrest: The first is based on the arrest of cell elongation, as defined by Eq. 3, and the second is based on the arrest of the septation process, as visualized by microscopy.

			Log Overabundance:			
Gene	Annotated gene function:	Message number: *μ*m (mRNA molecules/cell cycle)	TFNseq Replication log10 *o*	Imaging-based Elongation log10 *o*	Septation log10 *o*	(*N_C_*, *N*_P_)

*dnaA*	Regulation of replication initiation	30	0.02± 0.02	0.7 ± 0.1	0.0± 0.2	(4,4)
*dnaN*	Replication beta sliding clamp	49	1.5 ± 0.1	2.0 ± 3.0	1.4± 0.1	(134,8)
*ftsN*	Essential cell division/septation protein	20	2.6 ± 0.1	1.8 ± 0.2	0.6± 0.2	(19,5)
*murA*	Cell wall precursor synthesis	26	0.7 ± 0.5	1.1 ± 0.1	0.9± 0.2	(16,4)

## Data Availability

We include source data files and sequencing data from RNA-Seq experiments to quantify transcription levels. Gene Expression Omnibus (GEO) accession number TBA.
